# Endocannabinoids Differentially Modulate Synaptic Plasticity in Rat Hippocampal CA1 Pyramidal Neurons

**DOI:** 10.1371/journal.pone.0010306

**Published:** 2010-04-22

**Authors:** Jian-Yi Xu, Rongqing Chen, Jian Zhang, Chu Chen

**Affiliations:** Neuroscience Center of Excellence, School of Medicine, Louisiana State University Health Sciences Center, New Orleans, Louisiana, United States of America; INSERM U862, France

## Abstract

**Background:**

Hippocampal CA1 pyramidal neurons receive two excitatory glutamatergic synaptic inputs: their most distal dendritic regions in the stratum lacunosum-moleculare (SLM) are innervated by the perforant path (PP), originating from layer III of the entorhinal cortex, while their more proximal regions of the apical dendrites in the stratum radiatum (SR) are innervated by the Schaffer-collaterals (SC), originating from hippocampal CA3 neurons. Endocannabinoids (eCBs) are naturally occurring mediators capable of modulating both GABAergic and glutamatergic synaptic transmission and plasticity via the CB1 receptor. Previous work on eCB modulation of excitatory synapses in the CA1 region largely focuses on the SC pathway. However, little information is available on whether and how eCBs modulate glutamatergic synaptic transmission and plasticity at PP synapses.

**Methodology/Principal Findings:**

By employing somatic and dendritic patch-clamp recordings, Ca^2+^ uncaging, and immunostaining, we demonstrate that there are significant differences in low-frequency stimulation (LFS)- or DHPG-, an agonist of group I metabotropic glutamate receptors (mGluRs), induced long-term depression (LTD) of excitatory synaptic transmission between SC and PP synapses in the same pyramidal neurons. These differences are eliminated by pharmacological inhibition with selective CB1 receptor antagonists or genetic deletion of the CB1 receptor, indicating that these differences likely result from differential modulation via a CB1 receptor-dependent mechanism. We also revealed that depolarization-induced suppression of excitation (DSE), a form of short-term synaptic plasticity, and photolysis of caged Ca^2+^-induced suppression of Excitatory postsynaptic currents (EPSCs) were less at the PP than that at the SC. In addition, application of WIN55212 (WIN) induced a more pronounced inhibition of EPSCs at the SC when compared to that at the PP.

**Conclusions/Significance:**

Our results suggest that CB1 dependent LTD and DSE are differentially expressed at the PP *versus* SC synapses in the same neurons, which may have an impact on synaptic scaling, integration and plasticity of hippocampal CA1 pyramidal neurons.

## Introduction

Hippocampal CA1 pyramidal neurons receive two anatomically segregated glutamatergic synaptic inputs: their most distal dendritic regions in the stratum lacunosum-moleculare (SLM) receive direct sensory input from layer III neurons of the entorhinal cortex through the perforant path (PP) pathway, while their more proximal regions of the apical dendrites in the stratum radiatum (SR) receive information from hippocampal CA3 neurons through the Schaffer-collateral (SC) pathway. The information from the CA3 neurons through the SC originates from layer II neurons of the entorhinal cortex and is relayed through synapses onto granule neurons in the detate gyrus and pyramidal neurons in the CA3 region [1], [2]. The dual sensory inputs that the CA1 pyramidal neurons receive have been suggested to be essential for information processing, consolidation, storage and retrieval in the hippocampus [3]–[6]. For instance, it has been demonstrated that direct cortical input to CA1 through the PP pathway is required for long-term memory and plasticity [4], [6], [7]. LTP or LTD-inducing stimulation at the PP has been shown to modulate synaptic plasticity of the SC [5], [8], [9]. Recent evidence also shows that pairing of PP and SC inputs induces an input-timing-dependent plasticity of the SC in CA1 pyramidal neurons, indicating that interactions of distal PP and proximal synaptic inputs occur in the same CA1 pyramidal neurons [10].

Available evidence shows differences in expression and function of ion channels and receptors at synapses of CA1 pyramidal neurons between SLM and SR regions [4], [11]–[16]. The PP and SC also exhibit distinct synaptic responses to neurotransmitters or modulators [17]–[20], suggesting that synaptic activity at the PP and SC is differentially modulated by neurotransmitters. Endocannabinoids (eCBs), naturally occurring lipid mediators, are involved in a variety of physiological, pharmacological, and pathological processes [21]–[23]. While the role of eCBs as retrograde messengers in modulation of both GABAergic and glutamatergic synaptic activities in the CA1 region has been extensively investigated, little information is available on whether eCBs modulate synaptic transmission and plasticity at the CA1 PP pathway. Here, we report that there are differences in DHPG- and low-frequency stimulation (LFS)-induced LTD between the PP and SC synapses. These differences in LTD result likely from differentially modulation by a CB1 receptor-dependent mechanism in the same CA1 pyramidal neurons. Our results suggest that endocannabinoid modulation of glutamatergic synaptic transmission and plasticity is pathway-specific, which may have an impact on information processing and storage in hippocampal CA1 pyramidal neurons.

## Results

### eCB contribution to the difference in mGluR-induced LTD between the PP and SC

**Figure 1:**
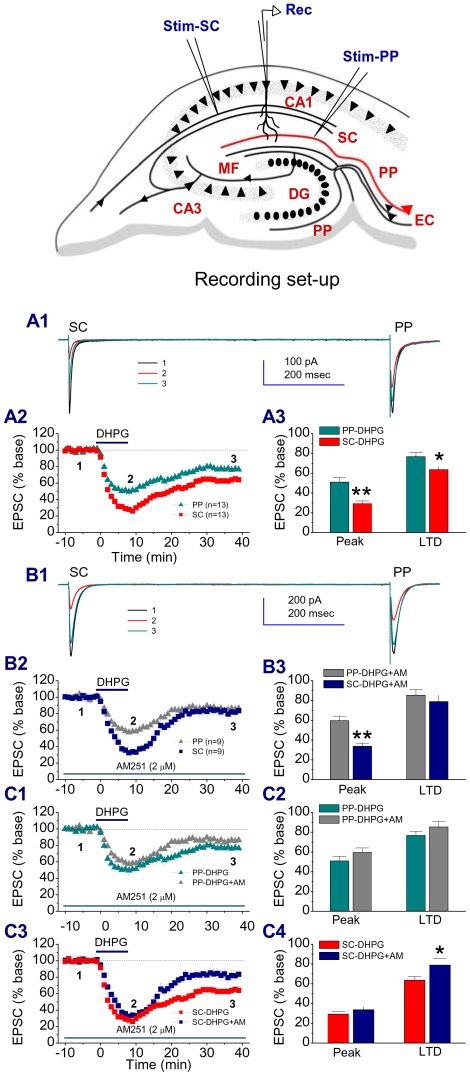
Endocannabinoid contribution to the difference in group 1 mGluR-induced LTD between the perforant path (PP) and Schaffer collateral (SC) pathways. Inset: recording setup. One stimulating electrode was placed in the site of the PP pathway, and another stimulating electrode was placed in the site of SC synapses. Somatic recordings under the whole-cell voltage clamp mode were made in CA1 pyramidal neurons. A1. Representative traces of EPSCs in response to independent PP and SC stimuli recorded from the same rat hippocampal CA1 pyramidal neuron in the absence and presence of DHPG (50 µM) and washout. A2. Time courses of DHPG-induced changes in EPSCs. A3. Mean values of EPSCs averaged from 6 to 10 (Peak) and 36 to 40 min (LTD) following DHPG application. *P<0.05, **P<0.01 compared with PP. B1. Representative traces of EPSCs recorded in slices treated with AM251 (2 µM) in the absence and presence of DHPG (50 µM) and washout. B2. Time courses of DHPG-induced changes in EPSCs in the presence of AM251. B3. Mean values of EPSCs averaged from 6 to 10 (Peak) and 36 to 40 min (LTD) following DHPG application. **P<0.01 compared with PP. C1. Time courses of DHPG-induced changes in EPSCs at PP in the absence and presence of AM251. C2. Mean values of PP EPSCs averaged from 6 to 10 (Peak) and 36 to 40 min (LTD) following DHPG application in the absence of presence of AM251. C3. Time courses of DHPG-induced changes in EPSCs at SC in the absence and presence of AM251. C4. Mean values of SC EPSCs averaged from 6 to 10 (Peak) and 36 to 40 min (LTD) following DHPG application in the absence of presence of AM251. *P<0.05 compared with DHPG.

Activation of the Group I metabotropic glutamate receptors (mGluRs) with a selective agonist, DHPG, has been shown to induce eCB-mediated LTD [24]–[27]. To determine whether eCBs are involved in DHPG-induced LTD of excitatory glutamatergic synaptic transmission at the PP and SC of rat hippocampal CA1 pyramidal neurons, we employed the whole-cell patch clamp recordings of Excitatory postsynaptic currents (EPSCs) in response to independent stimuli of the PP and SC of the same pyramidal neurons in the presence of DHPG. As seen in Figure 1A, bath application of DHPG (50 µM) for 10 min produced an acutely large depression and followed by a long-term depression of EPSCs. However, there are significant differences in DHPG-induced acute depression (peak) and LTD between the PP and SC pathways. To determine whether eCBs contributed to the DHPG-induced suppressions, we used AM251 (2 µM), a selective CB1 receptor antagonist. While AM251 did not prevent DHPG-induced peak suppression of EPSCs both at the PP and SC, antagonism of the CB1R reduced DHPG-induced LTD at the SC pathway (Fig. 1B). This reduction resulted in an elimination of the difference in the DHPG-induced LTD between the PP and SC. To confirm the effect of the inhibition of the CB1R on DHPG-mediated LTD, we used rimonabant (RIM), another selective CB1 receptor antagonist. As shown in supplementary Figure S1, RIM (2 µM) produced a similar effect as AM251 did, eliminating the difference in DHPG-induced LTD between the PP and SC. To determine whether the NMDA receptor is involved in DHPG-induced LTD at the SC, we included AP5 (50 µM), a selective NMDA receptor blocker, in the bath solution. As shown in supplementary Figure S2B, blockade of the NMDA receptor with AP5 did not affect DHPG-induced LTD at the SC, suggesting that mGluR activation-induced LTD at the SC does not require activation of the NMDA receptor.

**Figure 2:**
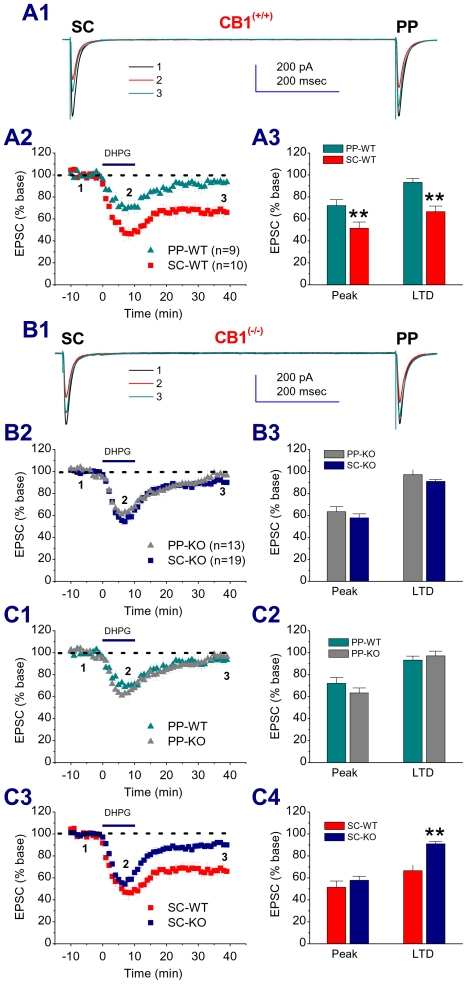
The difference in group 1 mGluR-induced LTD between the PP and SC pathways is absent in mice deficient in the CB1 receptor. A1. Representative traces of EPSCs recorded in hippocampal CA1 pyramidal neuron from a wild-type (WT) mouse in response to independent PP and SC stimuli in the absence and presence of DHPG (50 µM) and washout. A2. Time courses of DHPG-induced changes in EPSCs. A3. Mean values of EPSCs averaged from 6 to 10 (Peak) and 36 to 40 min (LTD) following DHPG application. **P<0.01 compared with PP. B1. Representative traces of EPSCs recorded in a mouse from a CB1R knockout (KO) mouse in the absence and presence of DHPG (50 µM) and washout. B2. Time courses of DHPG-induced changes in EPSCs. B3. Mean values of EPSCs averaged from 6 to 10 (Peak) and 36 to 40 min (LTD) following DHPG application. C1. Time courses of DHPG-induced changes in EPSCs at the PP in CB1R KO and their WT littermates. C2. Mean values of PP EPSCs averaged from 6 to 10 (Peak) and 36 to 40 min (LTD) following DHPG application. C3. Time courses of DHPG-induced changes in EPSCs at the SC in CB1R KO and their WT littermates. C4. Mean values of SC EPSCs averaged from 6 to 10 (Peak) and 36 to 40 min (LTD) following DHPG application. **P<0.05 compared with WT.

To further confirm the involvement of eCB signaling and its contribution to the difference in DHPG-induced LTD between the PP and SC, we used mice deficient in the CB1 receptor. Application of DHPG produced similar differences in acute depression and LTD between the PP and SC of hippocampal CA1 pyramidal neurons in wild-type (WT) mice (Fig. 2A), as observed in rat hippocampal neurons. As expected, the difference in DHPG-induced LTD between the PP and SC of CA1 pyramidal neurons was diminished in CB1R knockout (KO) mice (Fig. 2B). The results generated from CB1R antagonists and KO mice suggest that eCBs contribute to the DHPG-LTD at the SC, but not at the PP of CA1 pyramidal neurons.

### eCBs are involved in LFS-induced LTD at the SC of CA1 pyramidal neurons

**Figure 3:**
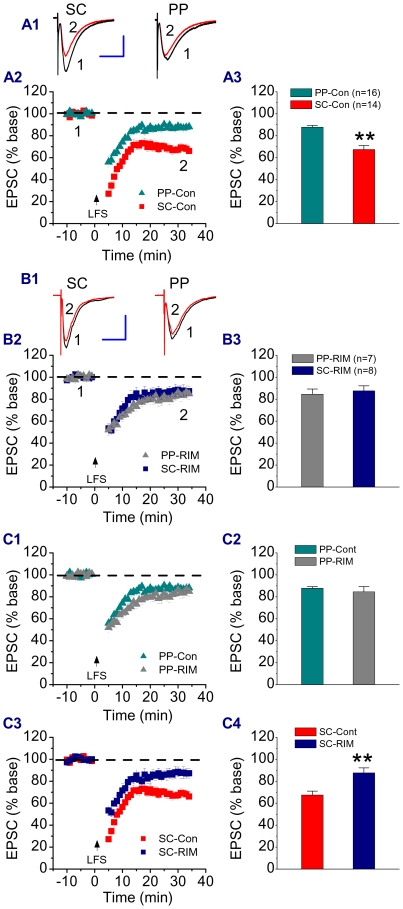
Endocannabinoids are involved in LFS-induced LTD at the SC of CA1 pyramidal neurons. A1. Representative traces of EPSCs recorded before and after LFS at the PP or SC in different rat hippocampal CA1 pyramidal neurons. LFS was delivered to the PP or SC independently. A2. Time courses of LSF-induced LTD at the PP and SC. A3. Mean values of LTD averaged from 30 to 35 min following LFS. **P<0.01 compared with LFS at the PP. B1. Representative traces of EPSCs recorded before and after LFS at the PP or SC of different rat hippocampal CA1 pyramidal neurons in slices treated with rimonabant (RIM, 2 µM). B2. Time courses of LSF-induced LTD at the PP and SC in the presence of RIM. B3. Mean values of LTD averaged from 30 to 35 min following LFS. C1. Time courses of LSF-induced LTD at the PP in the absence and presence of RIM. C2. Mean values of PP LTD in control and RIM. C3. Time courses of LSF-induced LTD at the SC in the absence and presence of RIM. C4. Mean values of SC LTD in control and RIM. **P<0.01 compared with SR. Scale bars: 200 pA/30 ms.

Previous studies demonstrated that eCBs participate in low-frequency stimulation (LFS)-induced LTD of excitatory synaptic transmission in the nucleus accumbens [28] and the cerebellum [29]. Recent evidence also shows that LTD in autaptic excitatory hippocampal neurons is CB1 receptor-dependent [30]. To determine whether eCBs are involved in LFS-induced LTD at the PP and SC of CA1 pyramidal neurons, LTD of excitatory synaptic transmission at the PP or SC was recorded in response to LFS at the PP or SC. While LTD was induced at the PP or SC in response to LFS, the magnitude of LTD at the PP was less pronounced when compared to that at the SC (Fig. 3A). Application of RIM (2 µM) reduced LTD at the SC, but not at the PP. The differences in the magnitude of LTD between the two pathways were eliminated by blockade of the CB1 receptor (Fig. 3B). These results indicate that eCBs are involved in SC LTD, but not PP. It is well accepted that LFS-induced LTD requires activation of the NMDA receptor. To ascertain whether LFS-induced LTD at SC in our recordings is NMDA receptor-dependent, we included AP5 (50 µM) in the bath solution. As shown in supplementary Figure S2A, LFS-induced LTD was blocked in the presence of AP5, suggesting that LFS-induced LTD at the SC synapse requires activation of the NMDA receptor. To see whether group I mGluRs are involved in the LFS-induced LTD at the SC, we used MPEP (20 µM) and LY367385 (50 µM), mGluR1/5 antagonists. Inhibition of mGluR1/5 did not prevent LFS-induced LTD at the SC (supplementary Figure S2A), indicating that group I mGluRs do not contribute to the LFS-induced LTD at the SC.

**Figure 4:**
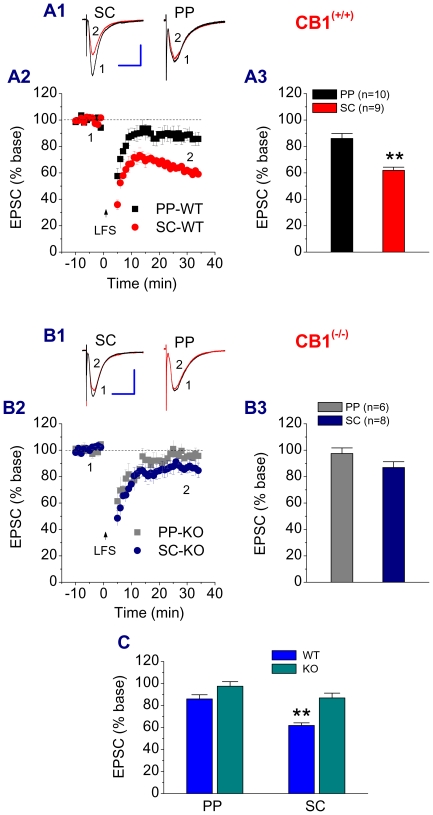
Removal of the CB1 receptor eliminates the difference in LTD between the PP and SC. A1. Representative traces of EPSCs recorded before and after LFS at the PP or SC in different hippocampal CA1 pyramidal neuron from WT. LFS was delivered to the PP or SC independently. A2. Time courses of LSF-induced LTD at the PP and SC in WT. A3. Mean values of LTD averaged from 30 to 35 min following LFS. **P<0.01 compared with LFS at the PP. B1. Representative traces of EPSCs recorded before and after LFS at the PP or SC of different rat hippocampal CA1 pyramidal neurons from CB1R KO. B2. Time courses of LSF-induced LTD at the PP and SC in CB1R KO. B3. Mean values of LTD averaged from 30 to 35 min following LFS. C. Summary for the differences in LFS-induced LTD at the SC and PP between WT and KO. **P<0.01 compared with KO.

To further confirm the contribution of eCBs to the difference in LTD between the two synapses, we used CB1R KO mice. As shown in Figure 4A, there was a difference in LTD between the PP and SC in WT animals. However, this difference was disappeared in CB1R KO mice (Fig. 4B), suggesting that the difference in LTD between the two pathways is attributed to a greater contribution eCBs in LTD at the SC, but lesser at the PP.

### Difference in DSE between the PP and SC

**Figure 5:**
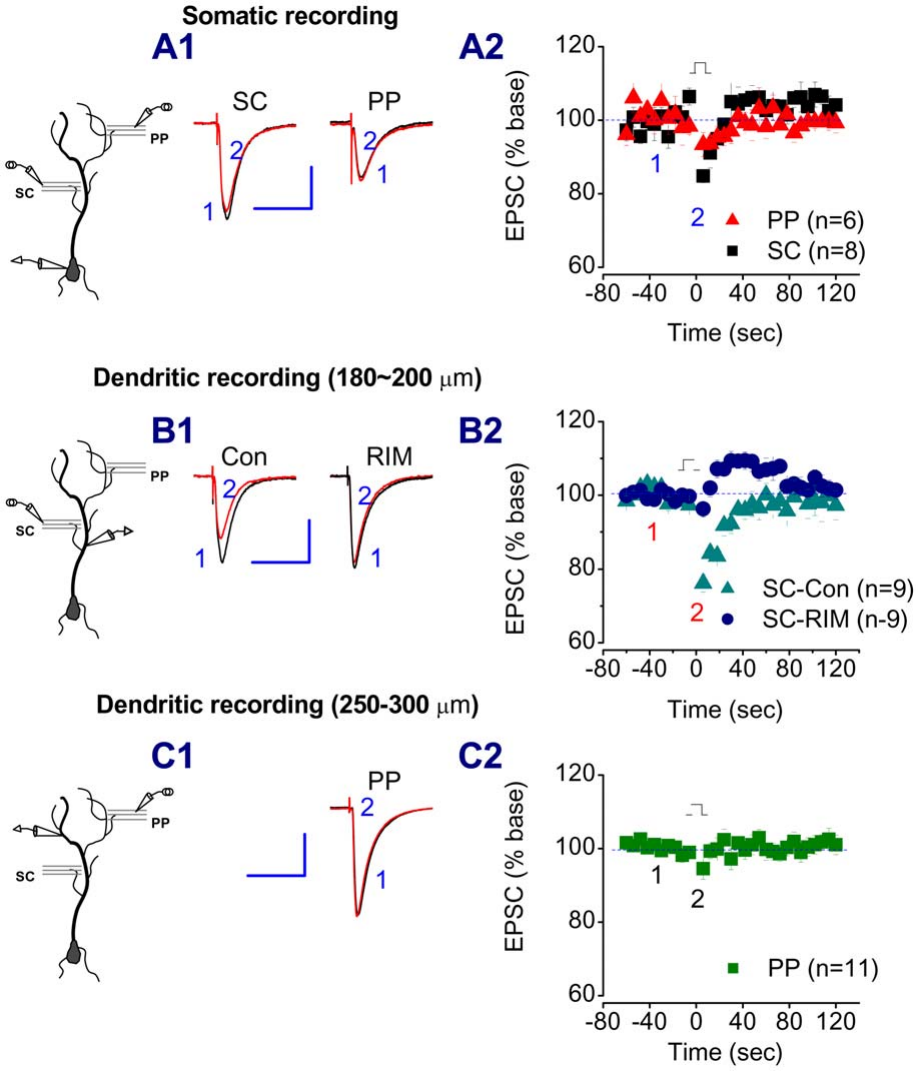
Depolarization-induced suppression of excitation (DSE) is present at the SC, but not at the PP. A1. Representative traces of EPSCs recorded before and after a somatic depolarizing step from −70 to 0 mV for a 10 sec duration at the PP and SC of a rat pyramidal neuron. A2. Time courses of DSE at the PP and SC. B1. Representative traces of EPSCs recorded before and after a dendritic (200 µm from the soma) depolarizing step from −70 to 0 mV for a 10 sec duration at the SC of pyramidal neurons in the absence and presence of RIM (2 µM). B2. Time courses of DSE induced by dendritic depolarization at the SC. C1. Representative traces of EPSCs recorded before and after a dendritic (280 µm from the soma) depolarizing step from −70 to 0 mV for a 10 sec duration at the PP of a pyramidal neuron. B2. Time courses of DSE induced by dendritic depolarization at the PP. Scale bars: 200 pA/50 msec.

Depolarization-induced suppression of inhibition (DSI) and depolarization-induced suppression of excitation (DSE) are eCB-mediated short term synaptic plasticity [22], [31]–[34]. Although depolarization-induced suppression of inhibition (DSE) is mainly seen at excitatory synapses in the cerebellum [35], DSE can also be induced in neurons of the hippocampus, except that a longer duration (e.g., 7–10 sec) of the depolarization step is required [36]. To determine whether there is a difference in eCB-mediated retrograde signalling in short-term synaptic plasticity between the PP and SC, DSE was recorded in pyramidal neurons by a 10 sec depolarizing step from a holding potential of −70 to 0 mV. As seen in Figure 5A, the depolarization of neurons by somatic recording only induced a small depression of EPSCs both at the PP (6.6±3.3%, n = 6) and the SC (15.2±1.8%, n = 8). The SC synapses on dendritic trees of the CA1 pyramidal neurons range from about 120 to 400 µm from the soma and the PP pathways are a further distance away from the soma. Because of the space-clamp problems [37], [38], it is likely that somatic depolarization may not be able to depolarize the dendrites (where the SC and PP synapses are located) to the desired potential (e.g., 0 mV). To solve this issue, dendritic recordings were made, as we described previously [39], [40]. As shown in Figure 5B, dendritic depolarization at distances between 180 to 200 µm from the soma resulted in increased DSE in response to SC stimulation (23.3±2.1%, n = 9, P<0.05 when compared to somatic depolarization). This depression was inhibited by RIM (3.7±2.2% of baseline, n = 9), suggesting that DSE is mediated via a CB1 receptor. To record DSE at the PP, dendritic recordings at distance between 250 to 300 µm from the soma were made. As shown in Figure 5C, dendritic depolarization did not increase DSE in response to PP stimulation (5.5±2.8% of baseline, n = 11).

**Figure 6:**
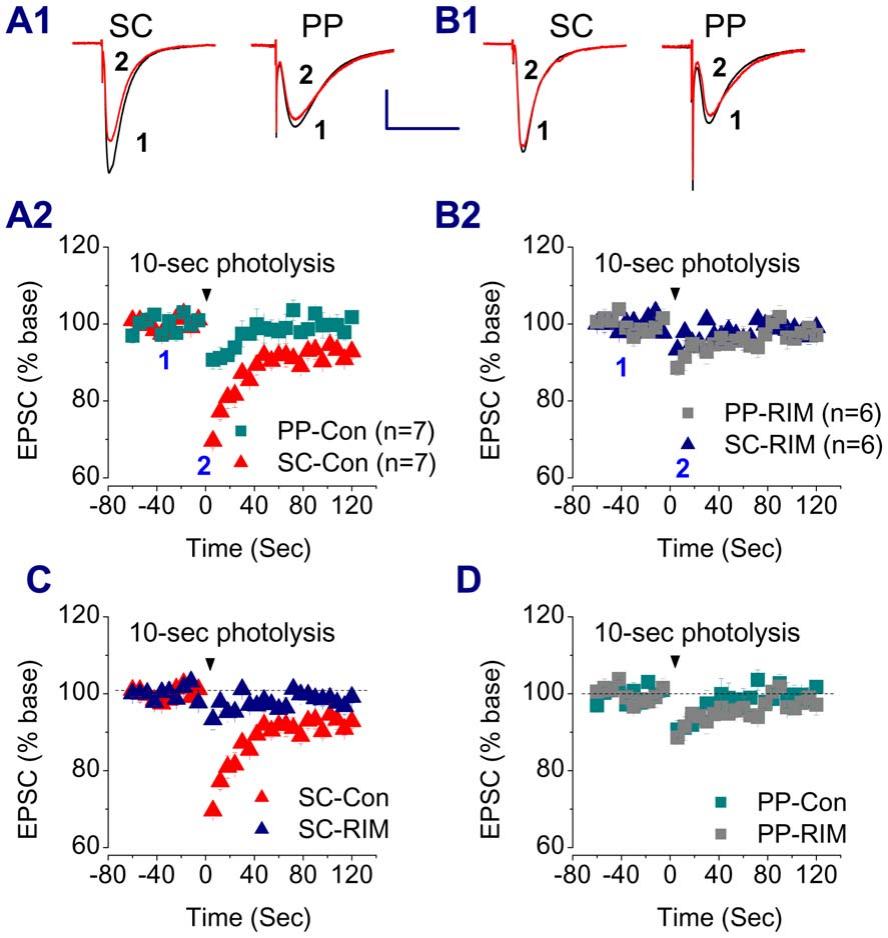
Photolysis of caged Ca^2+^-induced suppression of EPSCs at the PP and SC. A1. Representative traces of EPSCs recorded before and after a 10 sec photolysis of caged Ca^2+^ at the PP and SC of a rat pyramidal neuron. NP-EGTA (5 mM) was introduced into the cell via the recording patch pipette for dialysis at least 15 to 20 min before recordings were made. A2. Time courses of photolysis of caged Ca^2+^-induced suppression of EPSCs at the PP and SC. B1. Representative traces of EPSCs recorded before and after a 10 sec photolysis of caged Ca^2+^ at the PP and SC of a rat pyramidal neuron in the presence of RIM (2 µM). B2. Time courses of photolysis of caged Ca^2+^-induced suppression of EPSCs at the PP and SC in the presence of RIM. C. Time courses of photolysis of caged Ca^2+^-induced suppression of EPSCs at the SC in the absence and presence of RIM. D. Time courses of photolysis of caged Ca^2+^-induced suppression of EPSCs at the PP in the absence and presence of RIM. Scale bar: 200 pA/50 msec.

A rationale for using the depolarization protocol is to raise postsynaptic intracellular Ca^2+^, which triggers the release of eCBs in postsynaptic sites. However, the geometry and size of the distal dendrites prevented us from making dendritic recordings directly at the sites of the PP region. We therefore decided to increase intracellular Ca^2+^ in postsynaptic neurons by photolysis of caged Ca^2+^, as described previously [41], [42]. NP-EGTA (5 mM) was introduced into the cell via the recording patch pipette for dialysis at least 15 to 20 min. As indicated in Figure 6, a 10-sec UV flash produced transient inhibition of EPSCs at the SC (30.5±1%, n = 7, P<0.01 and P<0.05 when compared to somatic and dendritic depolarization, respectively) and at the PP (9.4±2.4%, n = 7). Photolysis of caged Ca^2+^-induced inhibition of EPSCs at the SC was blocked by RIM (6.7±2.7%, n = 6), but not at the PP (11.4±2.0%, n = 6), indicating that the small inhibition of EPSCs at the PP is eCB-independent. The results from DSE together with those from photolysis of caged Ca^2+^ suggest that there is virtually no DSE at the PP synapses of hippocampal CA1 pyramidal neurons, while DSE can be elicited at the SC of CA1 neurons via a Ca^2+^-dependent mechanism.

### Difference in response to application of exogenous cannabinoid between the PP and SC

**Figure 7:**
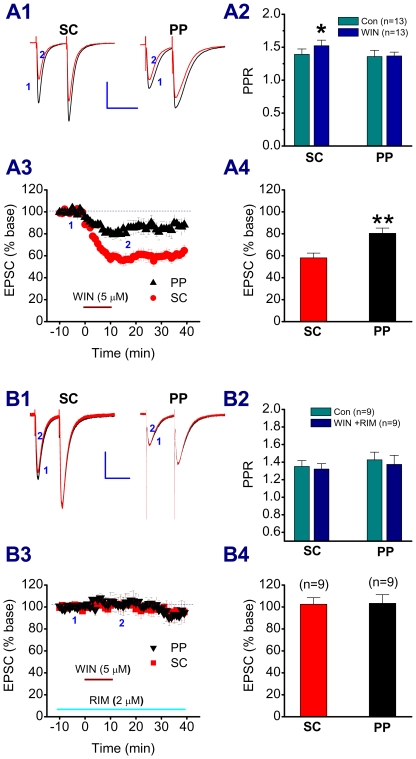
WIN55,212-2 produces different effects on EPSCs at the PP and SC. A1. Representative traces of paired-EPSCs recorded from a rat hippocampal CA1 pyramidal neuron in response to independent PP and SC stimuli in the absence and presence of WIN (5 µM). A2. Mean values of paired-pulse ratio (PPR) at the PP and SC in the absence and presence of WIN. A3. Time courses of WIN-induced changes in EPSCs at the PP and SC. A4. Mean values of EPSCs averaged from 20 to 25 min following WIN application. *P<0.05, **P<0.01 compared with PP. B1. Representative traces of paired-EPSCs recorded in slices treated with RIM (2 µM) in the absence and presence of WIN (5 µM). B2. Mean values of PPR at the PP and SC in the absence and presence of WIN+RIM. B3. Time courses of WIN-induced changes in EPSCs at the PP and SC in the presence of RIM. B4. Mean values of EPSCs averaged from 20 to 25 min following WIN application in RIM-treated slices. Scale bars: 200 pA/100 msec.

**Figure 8:**
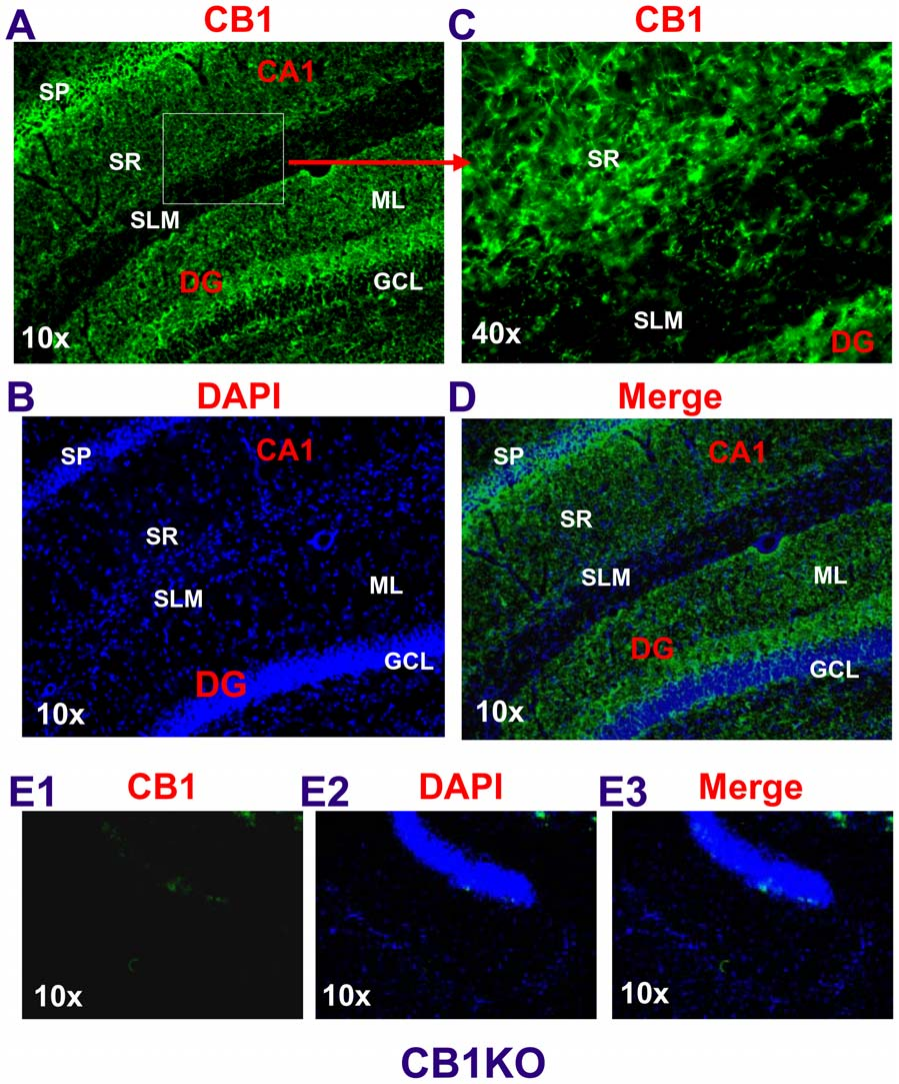
Expression of the CB1 receptor is relatively lower in the stratum lacunosum-moleculare (SLM) when compared to that in the stratum radiatum (SR). Rat or mouse hippocampal slices (30 µm) were prepared with cryostat, then cut and incubated with primary anti-CB1R antibody (1∶2,000) for 48 h at 4°C. Images were taken using a Zeiss deconvolution microscope. A–D. Immunostaining of rat hippocampal CB1 receptor. E1–E3. The specificity of the CB1 antibodies was determined by staining the slices from mice deficient in the CB1 receptor. SP: the stratum pyramidale; ML: molecular layer; GCL: granule cell layer; DG: dentate gyrus.

Based on the data showing differences in DHPG- and LFS-induced LTD, DSE, and photolysis of caged Ca^2+^-induced suppression of EPSCs between the PP and SC, it appears that there may be differences in the CB1 receptor distribution or density between the two pathways. To test this idea, we used a bath application of WIN55,212-2 (WIN), a potent synthetic cannabinoid receptor agonist exhibiting an inhibitory effect on both GABAergic and glutamatergic synaptic transmission in hippocampal CA1 pyramidal neurons [43]. As shown in Figure 7A, WIN (5 µM) produced an inhibition of EPSCs both at the PP and SC of the same pyramidal neurons. However, the magnitude of the inhibition was more pronounced at the SC when compared to that at the PP. Also, WIN increased paired-pulse facilitation ratio (PPR) at the SC, but not at the PP. The WIN-induced suppression of EPSCs was eliminated by RIM both at the PP and SC, suggesting that the inhibition is mediated via a CB1 receptor. This information indicates that there is a difference in expression and function of the CB1 receptor between the two synapses. To further confirm the differences in the expression of the CB1 receptor between SR and SLM, we performed immunostaining in hippocampal slices. As shown in Figure 8, there was a relatively lower immunoreactivity of the CB1 receptor in SLM when compared to that in SR. This is consistent with reports by others [33], [44]. Since the density of the CB1 receptor is higher at GABAergic terminals than that at glutamatergic terminals [33], it is possible that a strong CB1 staining signal in the SR may be associated with a higher density of the GABAergic synapses in this area. We also can not exclude the possibility that the increased intensity of CB1 receptor immunoreactivity in the SR *versus* SLM may be due to staining of glutamatergic terminals.

## Discussion

Endogenous cannabinoids as retrograde messengers modulate synaptic transmission and plasticity both at GABAergic and glutamatergic synapses via a CB1 receptor-dependent mechanism in the brain [21]–[23], [26], [33], [45], [46]. In the hippocampus, eCB modulation of synaptic activity focuses largely on GABAergic synapses [24], [32], [34], [47]. Accumulated information shows that eCBs play an important role in excitatory synaptic transmission and plasticity at the SC synapses of the hippocampal CA1 area through directly acting on CB1 receptors at glutamatergic terminals and indirectly influencing CB1 receptor activity at GABAergic terminals [24], [36], [47]–[51]. However, little is known about whether eCBs are important signalling mediators in modulating direct sensory input from the entorhinal cortex through the PP pathway in the hippocampal CA1 region. By employing whole-cell patch recording of EPSCs in the same CA1 pyramidal neurons in response to the PP or SC stimuli, we demonstrate that eCBs contribute to LFS- and group I mGluR activation-induced LTD of excitatory synaptic transmission at the SC, but not at the PP. The involvement of eCBs in short-term synaptic plasticity at the SC, but not the PP, is supported by the evidence that DSE and photolysis of caged Ca^2+^-induced suppression of EPSCs are present at the SC, but absent at the PP. The eCB-involved differential modulation of synaptic activities probably results from the differences in the extent of expression and function of the CB1 receptor at the two synapses. This is evident by the fact that application of WIN induced a smaller inhibition of EPSCs at the PP than that at the SC and that the CB1 immunoreactivity was lower in SLM than in SR.

Activation of group I metabotropic glutamate receptors (mGluRs) induces eCB-mediated inhibitory effects on both GABAergic and glutamatergic synaptic transmission [24], [25], [27], [52]–[54]. Group I mGluR-triggered and eCB-contributed LTD of inhibitory synapses has been described in several regions of the brain, including the cerebellum, striatum, hippocampus, and nucleus accumbens (see reviews: 25,26). While activation of group I mGluRs is capable of inducing LTD of excitatory synapses in the hippocampus [55]–[57], less is clear about whether eCBs are involved in this type of LTD in the hippocampus. We demonstrate here that DHPG, an agonist of the group I mGluRs, induces LTD at both PP and SC synapses. However, DHPG-induced LTD at the SC was significantly reduced by pharmacological inhibition or genetic deletion of the CB1 receptor, suggesting that eCBs contribute to the group I mGluR-induced LTD in the hippocampus. This is different from the previous reports where inhibition of the CB1 receptor did not induce a significant change in mGluR-induced LTD [58], [59]. The discrepancy between ours and other reports is still not clear, but this is presumably due to the differences in the concentration of DHPG used (50 µM in the present study versus 100 µM in others) and the ages of animals used (4 to 6 weeks in ours and P8-16 in [58]). The difference in the magnitude of DHPG-induced LTD between the PP and SC observed in the present study may not only be attributed to the differences in the extent of expression and function of mGluR_1/5_ between SLM and SR [13], [60], but also may result from the difference in the expression and function of the eCB system (eCBs, CBRs and enzymes synthesizing and degrading eCBs) between the two sub-regions [33].

Available information indicates that eCBs also participate in the LFS-induced LTD of excitatory synapses in the nucleus accumbens and cerebellum [28], [29], but little is known about the role of eCBs in hippocampal LTD. Recent evidence shows that LTD at the SC in autaptic excitatory hippocampal neurons is mediated via eCBs and dependent on the CB1 receptor [30]. While LTD at the SC is well described, LTD at the PP in the CA1 region is less documented. Earlier studies show that neurons in layer III of the entorhinal cortex primarily display low frequencies of firing; the neurons in this layer give rise to the PP pathway delivery of direct cortical information to distal dendrites in the CA1 [61], [62]. This means that LTD likely plays an important role in long-term plasticity at the PP of CA1 pyramidal neurons. In the present study, we confirm that LTD at the PP in the CA1 can be induced by LFS [63]. Even though LTD is modulated by eCBs at the SC pathway, it undergoes modulation to a much lesser extent at the PP pathway. This finding is consistent with the observations where eCBs are only involved in group I mGluR-induced LTD at the SC, but virtually not at all at the PP.

To further determine whether the differences in LFS- and DHPG-induced LTD between the PP and SC are associated with function of the eCB system, we first recorded DSE at the PP and SC of the CA1 pyramidal neurons. DSE at excitatory synapses of CA1 pyramidal neurons is not as easily induced as that of DSI [32], [34]. The duration of its depolarization step needs to be longer (e.g., 7–10 sec) in CA1 pyramidal neurons [36]. This is likely attributed to the space-clamp problems that are associated with somatic voltage-clamp recordings [37], [38]. To reduce space-clamp-induced errors, we made dendritic recordings, as we described previously [39], [40]. Our results indicate that dendritic depolarization significantly increases a CB1R-dpendent DSE at the SC and that photolysis of caged Ca^2+^ further enhances CB1R-dependent inhibition of EPSCs at the SC. However, there is virtually no DSE at the PP evident by dendritic recordings or photolysis of caged Ca^2+^, suggesting that there is less production of eCBs in distal dendrites of the pyramidal neurons in SLM, or lower expression of the CB1R on the PP pathway. Recent studies demonstrate that in the dentate gyrus, the lateral PP path, which originates from layer II neurons of the entorhinal cortex, exhibits small DSE and response to synthetic cannabinoid WIN [64]. Our results from exogenous application of WIN, which produces a smaller inhibitory effect on EPSCs at the PP than that at the SC, and from immunostaining, which shows a lower expression of the CB1R in SLM than that in SR, suggest that there is difference in functional expression of the CB1R between the two pathways in the CA1 region [33], [44].

Pyramidal neurons in the CA1 area receive direct sensory input through the PP pathway and indirect input through the trisynaptic pathways. The dual sensory inputs that the CA1 receive have been suggested to be essential for information processing, consolidation, storage and retrieval in the hippocampus [3]–[6]. It has been demonstrated that synaptic transmission at the PP and SC are differentially modulated by neurotransmitters or modulators. For instance, dopamine, serotonin and norepinephrine strongly inhibit field EPSPs at the PP in the CA1, but have little effect at the SC [18]–[20]. In contrast, carbachol, a cholinergic agonist, produces greater inhibition on fEPSPs at SR than that at the PP [17]. In the present study, we provide evidence that there are differences in DHPG- and LFS-induced LTD, DSE, and WIN-induced inhibition of EPSCs between the PP and SC in the same CA1 pyramidal neurons. This indicates that synaptic transmission and plasticity at the two pathways in the CA1 region are not only differentially modulated by monoamine and cholinergic modulators, but also by eCBs. Synaptic scaling, integration, and plasticity in CA1 pyramidal neurons are largely dependent on the inputs they receive and pre- and post-synaptic components (e.g., receptors, ion channels, and signaling molecules) that can be regulated or modulated by different neurotransmitters and modulators. The differential modulation of distal and proximal inputs by eCBs may play an important role in synaptic scaling, integration, and plasticity of CA1 pyramidal neurons and dialogue between the hippocampus and the entorhinal cortex. Disturbance or deficits in eCB modulation of hippocampal synaptic function can possibly lead to neurological or mental illnesses [65], [66]. Recent clinical trials of the CB1R antagonist, RIM (SR141716), for the treatment of obesity show a greater tendency toward depression and anxiety in patients taking RIM [67]–[69]. This means that there still are various previously undefined, complex eCB-mediated signaling pathways that warrant further investigation.

## Materials and Methods

### Hippocampal slice preparation

The care and use of the animals reported in this study were approved by the Institutional Animal Care and Use Committee of the Louisiana State University Health Sciences Center. Hippocampal slices were prepared from Sprague-Dawley rats (Charles River, Wilmington, MA) and CB1 knockout (KO) mice (cnr1^(−/−)^, NIMH transgenic core, NIH, Bethesda, MD) as described previously [39], [70], [71]. CB1R KO mice were backcrossed for more than ten generations onto the C57BL/6 background strain. Breeding of heterozygous mice produced homozygous CB1R wild type (WT) and KO mice. Age-matched littermates (either sex) were used in all the studies. Both rats and mice (12 to 16 days postnatal) were used for the experiments of low-frequency stimulation (LFS)-induced long-term depression (LTD), and rats and mice (4 to 6 weeks old) were used for the rest of the experiments. Briefly, after decapitation, brains were rapidly removed and placed in a cold oxygenated (95% O_2_, 5% CO_2_) low-Ca^2+^/high-Mg^2+^ slicing solution composed of (in mM) 2.5 KCl, 7.0 MgCl_2_, 28.0 NaHCO_3_, 1.25 NaH_2_PO_4_, 0.5 CaCl_2_, 7.0 glucose, 3.0 pyruvic acid, 1.0 ascorbic acid, and 234.0 sucrose. Slices were cut at a thickness of 400 µm and transferred to a holding chamber in an incubator containing oxygenated artificial cerebrospinal fluid (ACSF) composed of (in mM) 125.0 NaCl, 2.5 KCl, 1.0 MgCl_2_, 25.0 NaHCO_3_, 1.25 NaH_2_PO_4_, 2.0 CaCl_2_, 25.0 glucose, 3.0 pyruvic acid, and 1.0 ascorbic acid at 36°C for 0.5 to 1 h, and maintained in an incubator containing oxygenated ACSF at room temperature (∼22–24°C) for >1.5 h before recordings. Slices were then transferred to a recording chamber where they were continuously perfused with the 95% O_2_, 5% CO_2_-saturated standard ACSF at ∼32–34°C. Individual CA1 pyramidal neurons were viewed with a Zeiss Axioskop microscope (Oberkochen, Germany), fitted with a 60× (Olympus, Melville, NY) water-immersion objective and differential interference contrast (DIC) optics.

### Electrophysiological recordings

Whole-cell patch-clamp recordings were made using an Axopatch-200A patch-clamp amplifier (Molecular Devices, Sunnyvale, CA) under a voltage clamp. Pipettes (1–3 MΩ for somatic and 4–6 MΩ for dendritic recordings) were pulled from borosilicate glass with a micropipette puller (Sutter Instrument, Novato, CA). The internal pipette solution contained (in mM): 1) 130.0 KCH_3_SO_4_, 10.0 KCl, 10.0 HEPES, 0.1 EGTA, 4.0 Mg_2_ATP, 0.3 Tris_2_GTP, and 5.0 QX-314; or 2) 90.0 CsCH_3_SO_3_, 40.0 CsCl, 10.0 HEPES, 5.0 CaCl_2_, 4.0 Mg_2_ATP, 0.3 Tris_2_GTP, and 5.0 QX-314. The membrane potential was held at −70 mV. Excitatory postsynaptic currents (EPSCs) were recorded in response to stimuli of Schaffer collateral synapses (SC) in the stratum radiatum (SR) and perforant path synapses (PP) in the stratum lacunosum-moleculare (SLM) independently (500 to 800 ms apart) at a frequency of 0.05 Hz via bipolar tungsten electrodes or monopolar glass pipette electrodes filled with ACSF (Figure 1, inset). LTD at both SC and PP synapses was induced by DHPG, a group I metabotropic glutamate receptor (mGluR) agonist, or by an LFS protocol (900 stimuli at 3 Hz for 5 min at a holding potential of −40 mV). Paired-pulse stimulation was induced by delivering two pulses with an inter-pulse interval of 80–100 ms (Chen et al., 2001). Paired-pulse ratio (PPR) was calculated as P2/P1 (P1, the amplitude of the first EPSC; P2, the amplitude of the second EPSC). Depolarization-induced suppression of excitation (DSE) was elicited by a 10 sec depolarization from a holding potential of −70 to 0 mV. Bicuculline (10 µM) was added to the external solution to block GABAergic synaptic transmission. To ensure that the PP and SC are independently activated by electrical stimulation with electrodes placed at stratum radiatum (SR) and stratum lacunosum-moleculare (SLM), we used paired-pulses or tri-pulses to deliver stimuli at SC-PP or PP-SC. As shown in supplementary Figure S3A, paired pulses delivered to the same pathway, SC (SC-SC) or PP (PP-PP), produced a potentiation by the second EPSC. However, if the paired pulses were delivered to the PP and SC (PP-SC), no potentiation was seen at the second EPSC induced by stimulation at the SC. If a third stimulus was delivered to the SC following the second stimulus, a potentiation occurred at the third EPSC (supplementary Figure S3B2). The amplitude of the third EPSC at the SC was the same as that of the second EPSC when the first stimulus was delivered to the SC (supplementary Figure S3B1). A similar phenomenon was observed when the order of stimuli at the two pathways was reversed (supplementary Figure S3C1 & C2). Based upon our recordings, this information indicates that the PP and SC are independently activated by electrical stimulation.

### Photolysis of caged Ca^2+^

O-Nitrophenyl EGTA (NP-EGTA, Life Tech, Carlsbad, CA) was applied via the recording pipette. The internal pipette solution contained (in mM) 90.0 CsCH_3_SO_3_, 40.0 CsCl, 10.0 HEPES, 5.0 CaCl_2_, 4.0 Mg_2_ATP, 0.3 Tris_2_GTP, 5.0 QX-314, and 5.0 NP-EGTA. UV light from a Xenon arc flash lamp was delivered to the slice for 10 sec via the epifluorescence port of the microscope.

### Immunohistochemistry

Rats or mice (CB1R wild-type and knockout) were anesthetized with a ketamine/xylazine mixture (100/10 mg/kg, i.p.) and transcardially perfused with 4% paraformaldehyde in phosphate-buffered saline (PBS, pH 7.4). Brains were then immersed in 30% sucrose incubated at 4°C overnight for cryoprotection, and frozen in Tissue-Tex OCT reagent at −80°C. 30 µm-thick cryostat sections were cut and the sections were washed 3×10 minutes with PBS and blocked (5% normal goat serum, 2% BSA and 0.1% TritonX-100 in PBS) for 1 h. The sections were incubated with a primary polyclonal antibody to CB1R (1∶2,000, a kind gift of Dr. K. Mackie, Indiana University, or Cayman Chemical, Ann Arbor, Michigan, USA) at 4°C for 48 h, followed by incubation with fluorescently tagged secondary antibody for 1 h and DAPI for 10 min at room temperature, and then washed three times with PBS. The sections were mounted on slides and air dried overnight. Images were acquired using a Zeiss deconvolution microscope with the Slidebook 4.0 software.

Data were presented as mean ± S.E.M. Unless stated otherwise, Student's t-test and analysis of variance (ANOVA) with Student-Newman-Keuls test were used for statistical comparison when appropriate. Differences were considered significant when P<0.05.

## Supporting Information

Figure S1 Inhibition of the CB1 receptor eliminates the difference in DHPG-induced LTD between the PP and SC. Slices were treated with rimonabant (RIM, 2 µM). A1. Time courses of DHPG-induced changes in EPSCs at the PP and SC in the presence of RIM. A2. Mean values of EPSCs averaged from 6 to 10 (Peak) and 36 to 40 min (LTD) following DHPG application. **P<0.01 compared with PP. B1. Time courses of DHPG-induced changes in EPSCs at the PP in the absence and presence of RIM. C2. Mean values of PP EPSCs averaged from 6 to 10 (Peak) and 36 to 40 min (LTD) following DHPG application in the absence of presence of RIM. C1. Time courses of DHPG-induced changes in EPSCs at the SC in the absence and presence of RIM. C2. Mean values of SC EPSCs averaged from 6 to 10 (Peak) and 36 to 40 min (LTD) following DHPG application in the absence of presence of RIM. **P<0.01 compared with DHPG.
(0.57 MB TIF)

Figure S2 A. LFS-induced LTD at SC synapses requires activation of the NMDA receptor, but not group I mGluRs. AP5 (50 µM) or MPEP (20 µM) plus LY367385 (50 µM) were bath applied. A1. Time courses of LFS-induced LTD at the SC in the absence and presence of AP5 or MPEP+LY367385. A2. Mean values of LTD averaged from 36 to 40 min under conditions with different treatments. Blockade of the NMDA receptor eliminates LFS-induced LTD at SC synapses. B. DHPG-induced LTD at the SC does not require activation of the NMDA receptor. DHPG (50 µM) was bath applied for 10 min. B1. Time courses of DHPG-induced LTD in the absence and presence of AP5 (50 µM). B2. Mean value of LTD averaged from 36 to 40 min following application of DHPG in the absence and presence of AP5. mGluR activation-induced LTD at the SC does not require activation of the NMDA receptor.
(1.17 MB TIF)

Figure S3 Synapses at perforant path (PP) and Schaffer collateral (SC) in CA1 pyramidal neurons are independently activated by electrical stimulation. A 1 & A2. Representative traces recorded from a pyramidal neuron show that paired pulses delivered to the same pathway SC (SC-SC) or PP (PP-PP) induce a potentiation at second EPSC. B & C. Representative traces recorded from another two different pyramidal neurons show that paired pulses delivered to different pathways PP and SC (PP-SC) or SC and PP (SC-PP) do not induce a facilitation at second EPSC. B1. Paired pulses at SC-SC produce a potentiation at second EPSC. B2. There is no potentiation at second SC EPSC if the first stimulus was delivered to the PP. However, a third pulse is delivered to the SC induces a potentiation in the same neuron. C1. The first stimulus at the SC does not facilitate second EPSC-induced by stimulation at the PP, but the stimulus at the PP facilitates the third EPSC-induced by stimulation at the PP. C2. The order of stimuli at the two pathways is reversed in the same neuron as in C1.
(0.94 MB TIF)
