# Supplementary figures and images for: Endocannabinoids Differentially Modulate Synaptic Plasticity in Rat Hippocampal CA1 Pyramidal Neurons
Source: PLoS One. 2010 Apr 22;5(4):e10306. doi: 10.1371/journal.pone.0010306 (PMC2858667; doi:10.1371/journal.pone.0010306)

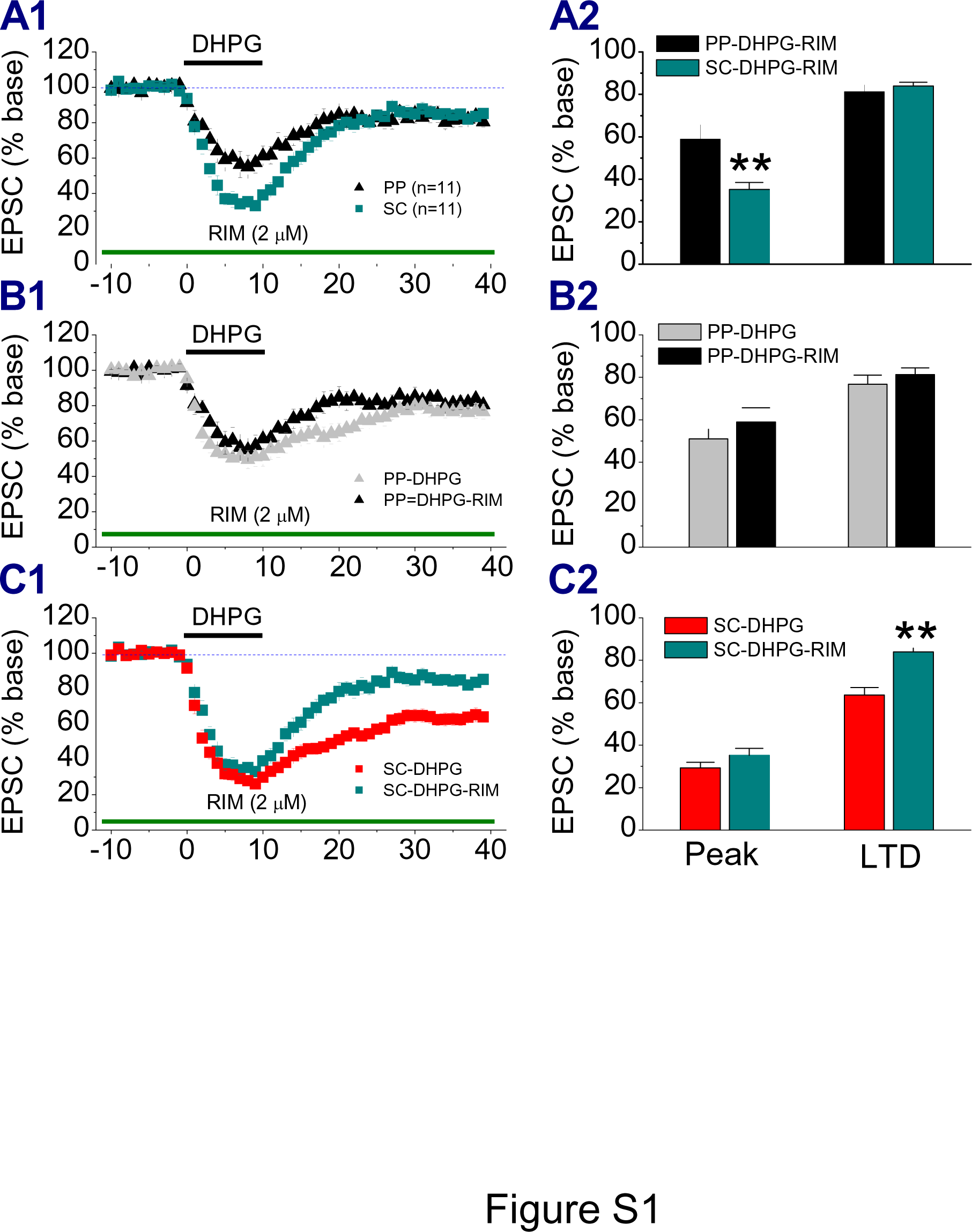

Supplement: Figure S1 — Inhibition of the CB1 receptor eliminates the difference in DHPG-induced LTD between the PP and SC. Slices were treated with rimonabant (RIM, 2 µM). A1. Time courses of DHPG-induced changes in EPSCs at the PP and SC in the presence of RIM. A2. Mean values of EPSCs averaged from 6 to 10 (Peak) and 36 to 40 min (LTD) following DHPG application. **P<0.01 compared with PP. B1. Time courses of DHPG-induced changes in EPSCs at the PP in the absence and presence of RIM. C2. Mean values of PP EPSCs averaged from 6 to 10 (Peak) and 36 to 40 min (LTD) following DHPG application in the absence of presence of RIM. C1. Time courses of DHPG-induced changes in EPSCs at the SC in the absence and presence of RIM. C2. Mean values of SC EPSCs averaged from 6 to 10 (Peak) and 36 to 40 min (LTD) following DHPG application in the absence of presence of RIM. **P<0.01 compared with DHPG. (0.57 MB TIF) [file pone.0010306.s001.tif]

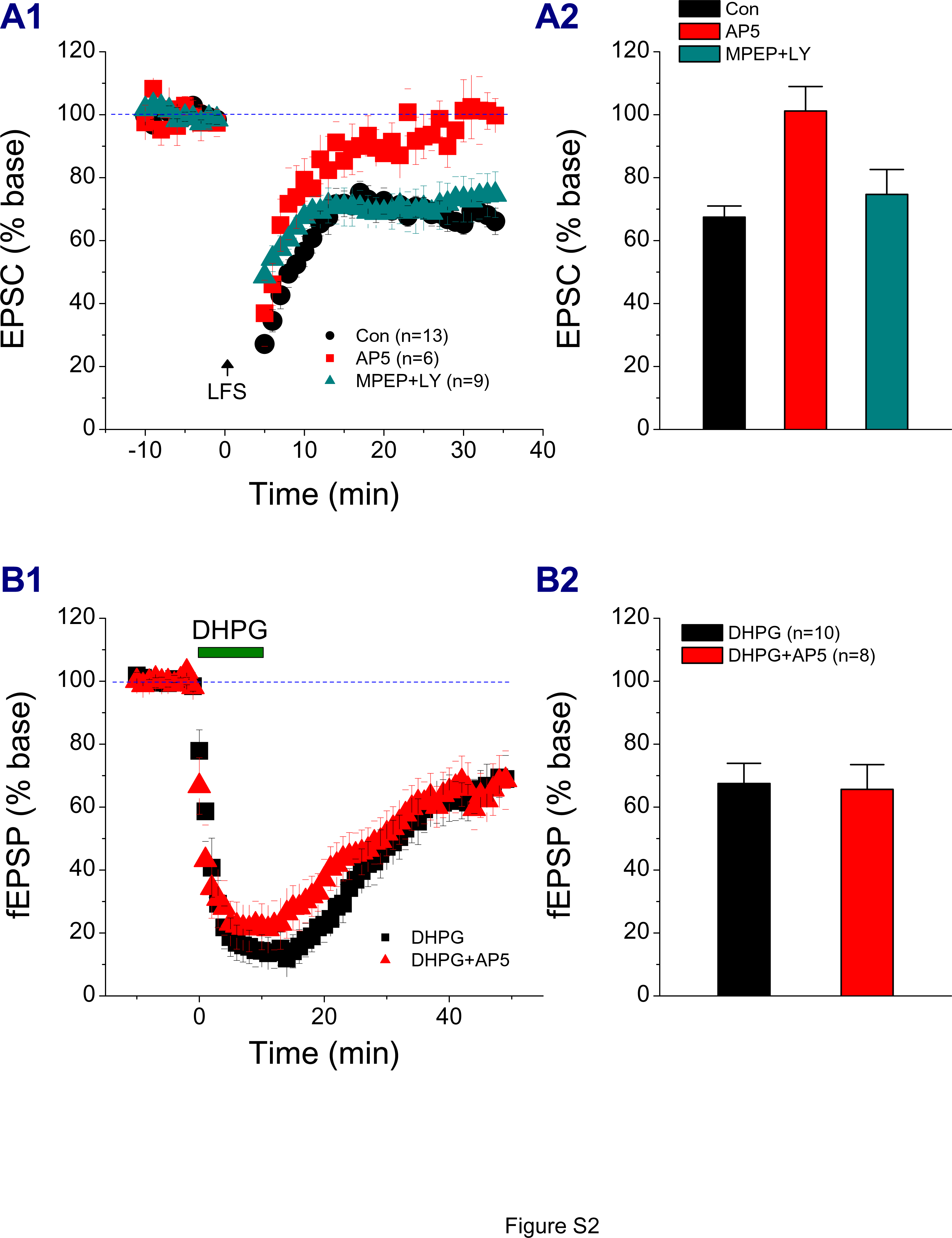

Supplement: Figure S2 — A. LFS-induced LTD at SC synapses requires activation of the NMDA receptor, but not group I mGluRs. AP5 (50 µM) or MPEP (20 µM) plus LY367385 (50 µM) were bath applied. A1. Time courses of LFS-induced LTD at the SC in the absence and presence of AP5 or MPEP+LY367385. A2. Mean values of LTD averaged from 36 to 40 min under conditions with different treatments. Blockade of the NMDA receptor eliminates LFS-induced LTD at SC synapses. B. DHPG-induced LTD at the SC does not require activation of the NMDA receptor. DHPG (50 µM) was bath applied for 10 min. B1. Time courses of DHPG-induced LTD in the absence and presence of AP5 (50 µM). B2. Mean value of LTD averaged from 36 to 40 min following application of DHPG in the absence and presence of AP5. mGluR activation-induced LTD at the SC does not require activation of the NMDA receptor. (1.17 MB TIF) [file pone.0010306.s002.tif]

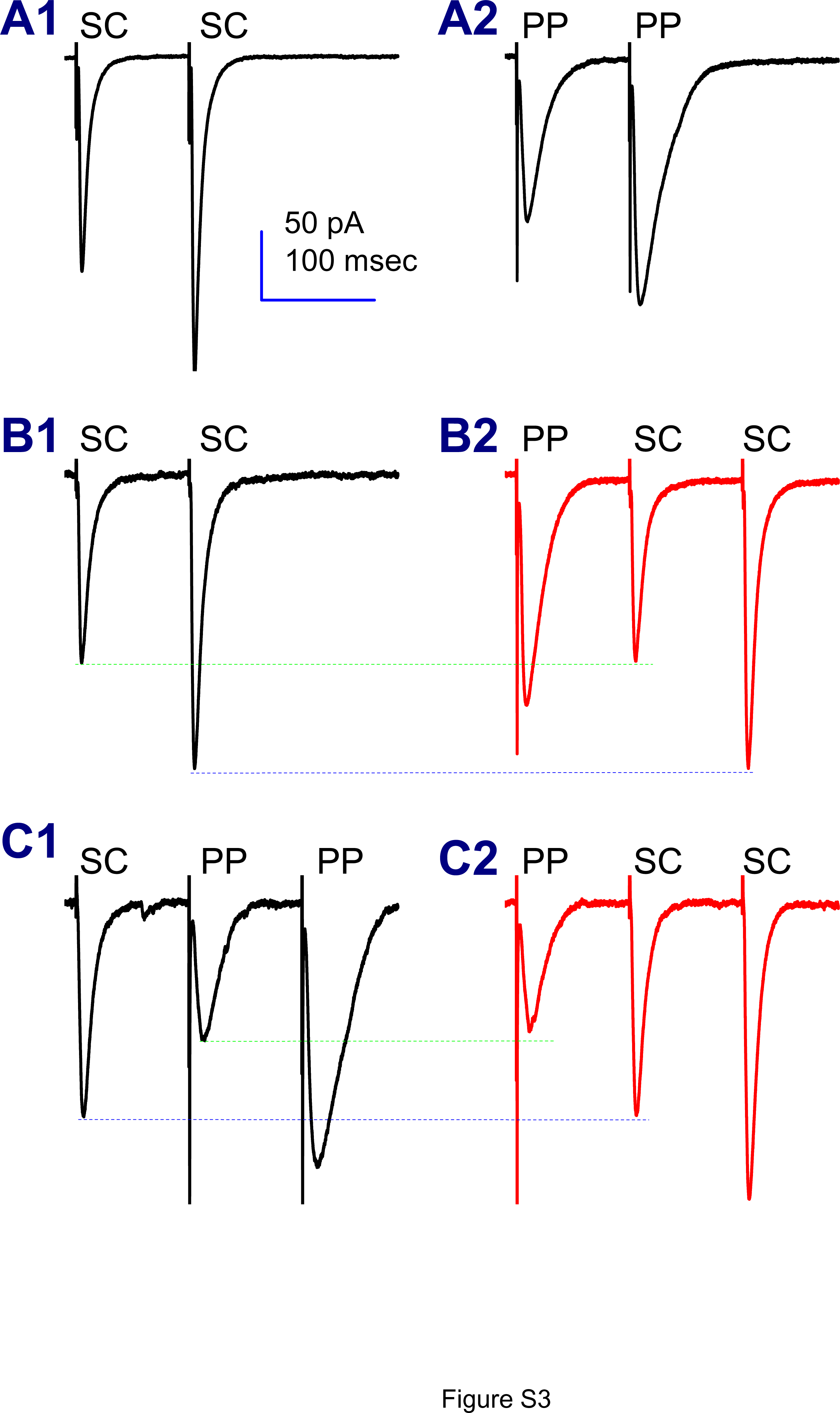

Supplement: Figure S3 — Synapses at perforant path (PP) and Schaffer collateral (SC) in CA1 pyramidal neurons are independently activated by electrical stimulation. A 1 & A2. Representative traces recorded from a pyramidal neuron show that paired pulses delivered to the same pathway SC (SC-SC) or PP (PP-PP) induce a potentiation at second EPSC. B & C. Representative traces recorded from another two different pyramidal neurons show that paired pulses delivered to different pathways PP and SC (PP-SC) or SC and PP (SC-PP) do not induce a facilitation at second EPSC. B1. Paired pulses at SC-SC produce a potentiation at second EPSC. B2. There is no potentiation at second SC EPSC if the first stimulus was delivered to the PP. However, a third pulse is delivered to the SC induces a potentiation in the same neuron. C1. The first stimulus at the SC does not facilitate second EPSC-induced by stimulation at the PP, but the stimulus at the PP facilitates the third EPSC-induced by stimulation at the PP. C2. The order of stimuli at the two pathways is reversed in the same neuron as in C1. (0.94 MB TIF) [file pone.0010306.s003.tif]
